# The N-terminal region of influenza virus polymerase PB1 adjacent to the PA binding site is involved in replication but not transcription of the viral genome

**DOI:** 10.3389/fmicb.2013.00398

**Published:** 2013-12-18

**Authors:** Nguyen Trong Binh, Chitose Wakai, Atsushi Kawaguchi, Kyosuke Nagata

**Affiliations:** Department of Infection Biology, Faculty of Medicine and Graduate School of Comprehensive Human Sciences, University of TsukubaTsukuba, Japan

**Keywords:** influenza virus, promoter binding, replication, reverse-genetics, RNA-dependent RNA polymerase, transcription

## Abstract

The influenza virus genome forms viral ribonucleoprotein (vRNP) complexes with nucleoprotein and viral RNA-dependent RNA polymerases (RdRp), PB1, PB2, and PA subunits. The vRNP complex catalyzes both genome replication and transcription reactions. PB1 contains the motifs highly conserved among RdRps and functions as a catalytic subunit of RdRp. The N-terminal region of PB1 between amino acid (a.a.) positions 1–83 contains both putative vRNA and cRNA promoter binding sites and a PA binding site. However, except for the PA binding site, the crystal structure and the function of the N-terminal region of PB1 are poorly understood. Here, we have examined the functional structure of the N-terminal region of PB1. The regions between a.a. positions 1–50 are highly conserved between influenza A and B viruses, but amino acids at positions 16, 27, and 44 are different between two viruses. To elucidate the functional importance of these amino acids in replication and transcription of the viral genome, we generated viruses containing mutations at these positions by reverse genetics and examined replication and transcription activities of these mutants. We found that a.a. positions 27 and 44 are responsible for the viral replication activity but not transcription activity.

## Introduction

Influenza A and B viruses contain eight-segmented and negative-stranded RNAs (vRNA) as its genome. Each segment is encapsidated by nucleoprotein (NP) and associated with viral RNA-dependent RNA polymerases (RdRp) to form viral ribonucleoprotein (vRNP) complexes. The vRNP complex is a basic unit for both genome replication and transcription (Nagata et al., 2008).

**Figure 1:**
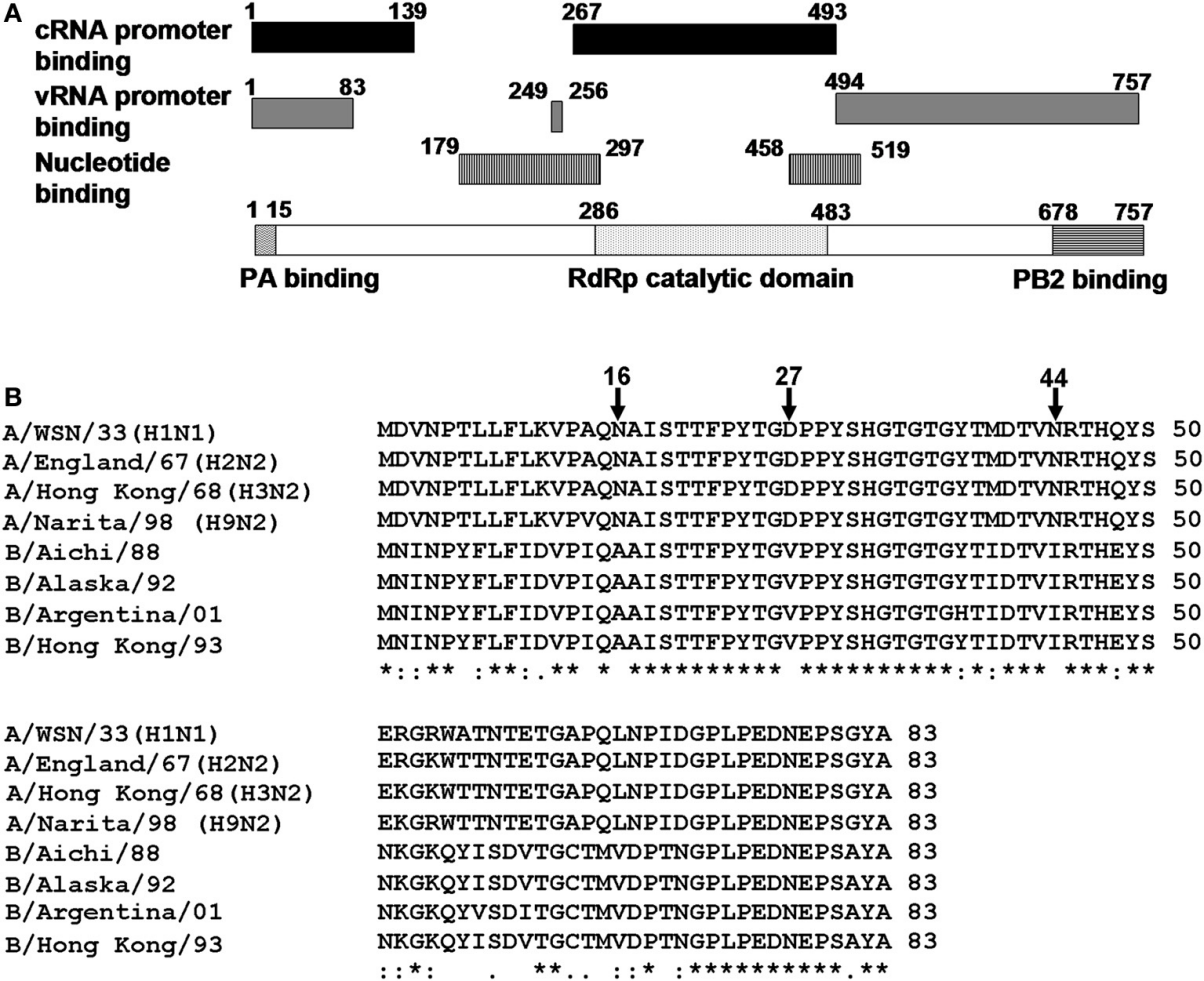
**Structure of PB1 subunit. (A)** A diagrammatic representation of PB1. Black bar, cRNA promoter binding sites; gray bar, vRNA promoter binding sites; black and white vertical stripes, nucleotide binding sites; black and white horizontal stripes, PB2 binding site; black waved lines, PA binding site, and black dots, RNA dependent-RNA polymerase catalytic domain. **(B)** Alignment of amino acid sequences of putative RNA binding region common to vRNA and cRNA promoters (1–83 a.a.) among influenza A and influenza B viruses. PB1 sequences of A/WSN/33, A/WSN/1933 (H1N1); A/England/67, A/England/10/67 (H2N2); A/Hong Kong/68, A/Hong Kong/1/1968 (H3N2); A/Narita/98, A/parakeet/Narita/92A/98 (H9N2). These strains are picked up from different clades and periods of PB1 gene phylogenetic tree (Taubenberger et al., 2005); B/Aichi/88, B/Aichi/5/88; B/Alaska/92, B/Alaska/03/1992; B/Argentina/01, B/Argentina/132/2001; and B/Hong Kong/93, B/Hong Kong/02/1993. Sequences were aligned with CLUSTAL W. Asterisk (^*^), identical residues; colon (:), conserved residues, and dot (.), semi-conserved residues.

The viral RdRp is a heterotrimer consisting of PB1, PB2, and PA subunits. Among them, PB1 functions as a catalytic subunit and assembly core of RdRp (Biswas and Nayak, 1996; Gonzalez et al., 1996; Toyoda et al., 1996; Zurcher et al., 1996; Ohtsu et al., 2002). The crystal structure of the interaction domains between N-terminal region of PB1 and C-terminal region of PA, and between C-terminal region of PB1 and N-terminal region of PB2 were resolved (He et al., 2008; Obayashi et al., 2008). PB1 contains the motifs highly conserved among RdRps, putative nucleotide-binding sites, and vRNA and cRNA promoter binding sites (Asano and Ishihama, 1997; Li et al., 1998; Gonzalez and Ortin, 1999a,b; Kolpashchikov et al., 2004) (Figure 1A).

The N-terminal region of PB1 (1–83 a.a.) contains both putative vRNA and cRNA promoter binding sites. However, except for the PA binding site (1–15 a.a.), the function of this region was poorly understood. Alignment of amino acid sequences revealved that the a.a. positions 1–50 was highly conserved between influenza A and B viruses except for the amino acid positions 16, 27, and 44. To identify the functional importance of these positions for the viral RNA synthesis, we determined the replicational and transcriptional activities using mutant viruses. Our results strongly suggest that the a.a. positions 27 and 44 are involved in replication process but not transcription process.

## Materials and methods

### Biological materials

Monolayer cultures of 293T and MDCK cells were maintained at 37°C with 5% CO_2_ in Dulbecco's Modified Eagle Medium (DMEM) and minimal essential medium (MEM) (Nissui), respectively, supplemented with 10% fetal bovine serum (Bovogen). Influenza virus strain A/WSN/33 (WSN) was prepared as previously described (Kawaguchi et al., 2005). Cycloheximide (CHX) was purchased from Sigma-Aldrich.

### Generation of recombinant viruses

To construct plasmids from which human DNA-dependent RNA polymerase I (Pol I) transcribes mutated vRNAs, we amplified fragments containing mutated segment 2 by PCR using a plasmid containing wild type WSN segments in pHH21 vector (Neumann et al., 1999) as a template with sets of phosphorylated primers (see Table S1 in the supplemental material). The amplified PCR products were self-ligated followed by sequencing. To generate recombinant viruses containing viral RNAs of WSN and mutated segment 2, reverse genetics system was used as described previously (Neumann et al., 1999). After 48 h post transfection (hpt), aliquots of cell culture supernatants were used for virus amplification in MDCK cells. At 48 h post infection (hpi), the culture fluid was collected, and the virus titer of these recombinant viruses was determined by plaque assays.

### RNA analysis by qRT-PCR

MDCK cells were infected with recombinant viruses at the multiplicity of infection (MOI) of 2.5. At 9 hpi, total RNA was isolated by the acid guanidine-phenol-chloroform method. To measure the accumulation levels of viral mRNA, cRNA, and vRNA, quantitative RT-PCR (qRT-PCR) was performed. Total RNAs were subjected to reverse transcription using ReverTraAce (Toyobo) with either (i) oligo (dT)_20_, (ii) 5′-AGTAGAAACAAGGGTATTTTTCTTTA-3′, or (iii) 5′-GACGATGCAACGGCTGGTCTG-3′ for synthesizing cDNA from segment 5 mRNA, cRNA, and vRNA, respectively (Kawaguchi and Nagata, 2007; Sugiyama et al., 2009). The synthesized single-stranded cDNAs were subjected to real-time quantitative PCR analysis (Thermal Cycler Dice real-time system TP800; TaKaRa) with SYBR Premix Ex *Taq* (TaKaRa) and a set of specific primers for segment 5 cDNA (see supplementary methods). The levels of these RNAs were normalized by the amount of cellular β-actin mRNA measured using specific primers (see supplementary methods). These results are averages from three independent experiments with standard deviations. The level of significance was determined by Student's *t*-test (unpaired).

## Results

### RNA synthesis of influenza A mutant viruses containing influenza B virus-type amino acid signatures

**Figure 2:**
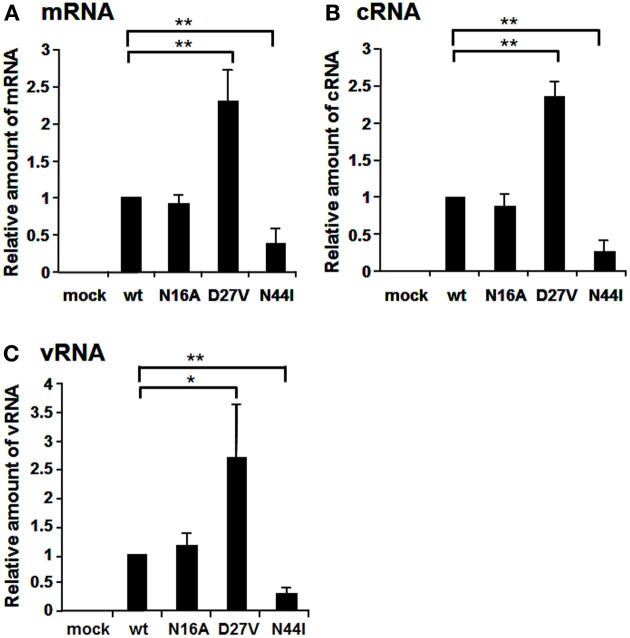
**Comparison of RNA synthesis of mutant influenza A viruses containing amino acids specific for influenza B viruses**. The amino acids, N, D, and N at the positions 16, 27, and 44 were mutated to A, V, and I, respectively. MDCK cells were infected with mutant viruses at MOI of 2.5. At 9 hpi, the accumulation levels of viral mRNA **(A)**, cRNA **(B)**, and vRNA **(C)** were measured by qRT-PCR, and the amounts of these RNAs were normalized by that of cellular actin mRNA. These results are averages from three independent experiments with standard deviations. ^*^p < 0.05; ^**^p < 0.01.

The N-terminal region of PB1 (1–83 a.a.) contains the PA binding site and both putative vRNA and cRNA promoter binding sites (Figure 1A). It is shown by alignment of amino acid sequences that the PB1 region between a.a. positions 1–50 are highly conserved between influenza A and B viruses, while the region between a.a. positions 51–83 differ between two viruses (Figure 1B). In the highly conserved region, except for the PA binding site, a.a. at positions 16, 27, and 44 are different between these viruses. Furthermore, these a.a. positions in PB1 are conserved more than 99% of influenza A and B viruses deposited in the NCBI Influenza Virus Sequence Database (Table 1). To elucidate the functional importance of these a.a. for viral RNA synthesis, we generated influenza A viruses containing Ala at the a.a. position 16 (N16A), Val at the a.a. position 27 (D27V), and Ile at the a.a. position 44 (N44I) by reverse genetics. We examined the RdRp activity by measuring the accumulation levels of viral mRNA, cRNA, and vRNA by qRT-PCR (Figure 2). The levels of all three type RNAs from D27V were increased compared with those from wild type and N16A virus, while those from N44I were significantly decreased. Based on the result that mutations at the positions 27 and 44 affect the synthesis activity of all viral RNAs equally, there could be two possibilities: (i) these mutations affect on the vRNA promoter recognition and followed by cRNA/mRNA synthesis, but do not affect on the cRNA promoter recognition and followed by vRNA synthesis, or (ii) these mutations affect independently the synthesis of each viral RNA, but total effects leads similar outputs in the synthesis of all viral RNAs.

**Table 1:** **Conservation of amino acid position 16, 27, and 44 in PB1**.

**Virus type**	**a.a. position**	**Mutation**	**No. of strains**	**Percentage**
			**(total strains)**	
**INFLUENZA A VIRUS**				
	16	N (wild type)	7228 (7259)	99.6%
		S	24 (7259)	0.3%
		D	4 (7259)	0.1%
		K	1 (7259)	0.0%
		H	1 (7259)	0.0%
		Y	1 (7259)	0.0%
	27	D (wild type)	7250 (7259)	99.9%
		N	4 (7259)	0.1%
		E	3 (7259)	0.0%
		G	2 (7259)	0.0%
	44	N (wild type)	7204 (7259)	99.2%
		S	42 (7259)	0.6%
		T	11 (7259)	0.2%
		D	2 (7259)	0.0%
**INFLUENZA B VIRUS**				
	16	A (wild type)	1412 (1412)	100%
	27	V (wild type)	1412 (1412)	100%
	44	I (wild type)	1409 (1412)	99.8%
		V	3 (1412)	0.2%

The conservation of amino acid position 16, 27, and 44 in PB1 was calculated by using 7259 sequences of human and avian influenza A strains and 1412 sequences of influenza B strains listed at the NCBI Influenza Sequence Database.

**Figure 3:**
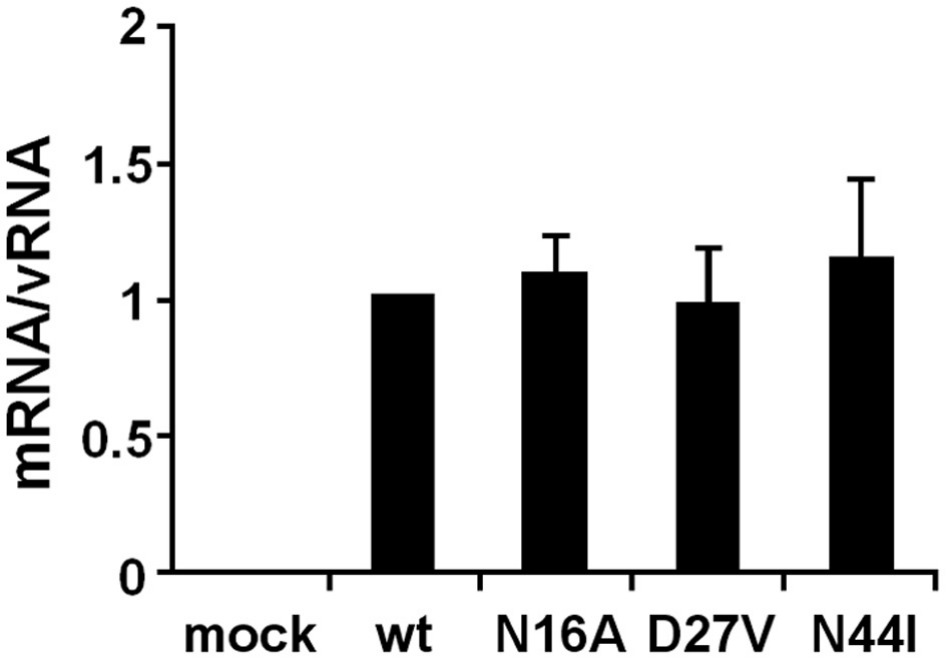
**Primary transcription activity of mutant influenza A viruses containing amino acids specific to influenza B viruses**. MDCK cells were infected with mutant viruses at MOI of 2.5 and incubated in the presence of 1.0 μg/ml of CHX. At 9 hpi, the accumulation levels of viral mRNA and vRNA were measured by qRT-PCR, and the amounts of these RNAs were normalized by that of cellular actin mRNA. The transcription activity is represented as a ratio of the amount of viral mRNA to that of vRNA. These results are averages from three independent experiments with standard deviations.

To elucidate whether these mutations affect genome replication (cRNA and vRNA synthesis) and/or transcription (viral mRNA synthesis) activities, we measured the primary transcription activity using cycloheximide (CHX), a potent inhibitor of protein synthesis (Figure 3). It is shown that CHX suppresses viral protein synthesis and thereby leads to degradation of replicated virus genome RNA but not viral mRNA since newly vRNP formation was repressed (Vreede et al., 2004; Kawaguchi et al., 2005). We utilized this method to measure the primary transcription activity that depends just only on incoming vRNP and is not affected by the replication process. In the presence of CHX, the levels of viral mRNA and vRNA were measured by qRT-PCR, and the transcription activity was represented as a ratio of viral mRNA/vRNA. This result shows that the transcription activity is not affected by these mutations, and thereby strongly suggests that these mutations affect the replication activity.

### Amino acid properties at a.a. positions 27 and 44 for the RNA synthesis activity

**Figure 4:**
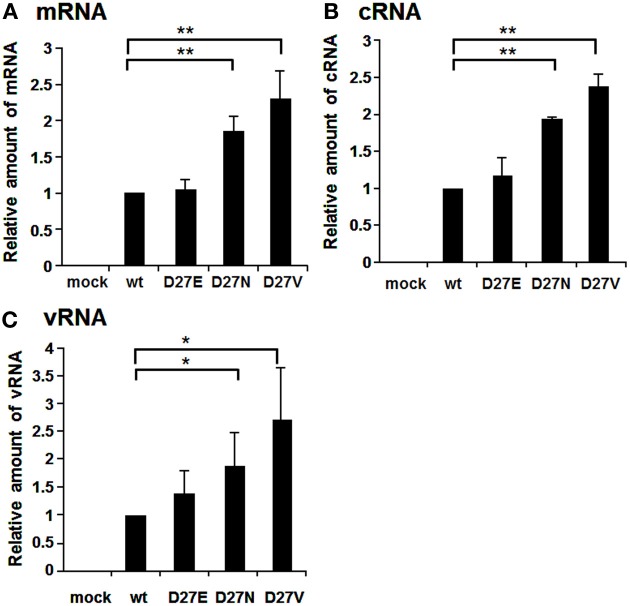
**RNA synthesis of viruses containing mutations at the amino acid position 27**. Wild-type virus and mutant viruses containing amino acids D and E, N, and V, respectively, at the a.a. position 27 were infected into MDCK cells at MOI of 2.5. At 9 hpi, the accumulation levels of viral mRNA **(A)**, cRNA **(B)**, and vRNA **(C)** were measured by qRT-PCR, and the amounts of these RNAs were normalized by that of cellular actin mRNA. These results are averages from three independent experiments with standard deviations. ^*^p < 0.05; ^**^p < 0.01.

Aspartate at the position 27 is highly conserved among influenza A viruses, except for an H4N8 strain isolated from least sandpiper that contains asparagine (GenBank: ACI90144.1). We generated D27E and D27N in addition to D27V (Figure 4). The RNA levels of mRNA, cRNA, and vRNA of D27N and D27V were increased significantly compared with those of wild-type and D27E. One of possible interpretations is that uncharged amino acids at a.a. position 27 may enhance the RNA synthesis.

**Figure 5:**
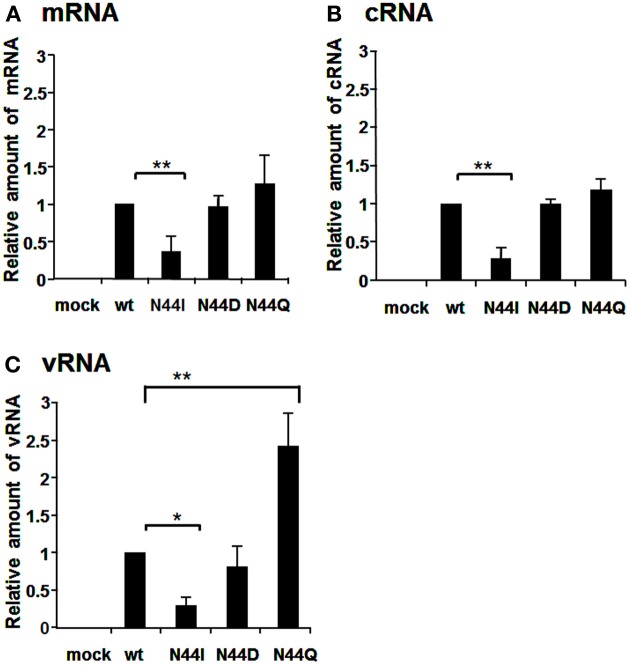
**RNA synthesis of viruses containing mutations at the amino acid position 44**. Wild-type virus and mutant viruses containing amino acids N and I, D, and Q, respectively, at the a.a. position 44 were infected into MDCK cells at MOI of 2.5. At 9 hpi, the accumulation levels of viral mRNA **(A)**, cRNA **(B)**, and vRNA **(C)** were measured by qRT-PCR, and the amounts of these RNAs were normalized by that of cellular actin mRNA. These results are averages from three independent experiments with standard deviations. ^*^p < 0.05; ^**^p < 0.01.

The mutation at the a.a. position 44 reduced the replication activity (Figure 2). To clarify the importance of the a.a. at this position, we additionally generated N44D and N44Q viruses in addition to N44I and examined the RNA synthesis activity (Figure 5). The synthesis level of each viral RNA of N44I was decreased largely, while the amounts of mRNA and cRNA of N44D and N44Q were similar to those of wild type. In addition, the amount of vRNA of N44Q was more than that of wild type. Thus, it is expected that the a.a. position 44 might be a water-soluble characteristic, and especially glutamine at this position stimulates the vRNA synthesis.

## Discussion

In this report, we have studied on three a.a. positions, i. e., 16, 27, and 44, which are not conserved between influenza A and B viruses. The RNA synthesis activity of D27V was enhanced, while that of N44I was decreased (Figure 2). Based on these, we carried out further mutational analyses. The N44I showed the decreased level of RNA synthesis in three types of viral RNAs, while N44D did not affect the RNA synthesis (Figure 5). Interestingly, N44Q increased vRNA synthesis with little effect on viral mRNA and cRNA synthesis. It is possible that side chain group of Q may stimulate the cRNA promoter binding and increase the vRNA synthesis activity.

D27V and D27N increased the RNA synthesis, while D27E mutation gave no effects (Figure 4). Furthermore, when the amounts of RNAs of D27V were analyzed at various MOI, those of D27V were increased (Figure S3). Although uncharged amino acid at this position enhances the RNA synthesis, molecular evolution has selected negatively charged amino acids. Therefore, it is assumed that charged amino acids at this position, even with low efficiency for the replication, are needed for PB1. Recently, mutational analyses showed that the sequences surrounding the PB1 AUG codon are multifunctional, and contain overlapping signals for translation initiation and for segment specific packaging (Wise et al., 2011). We may consider a possibility that there is some regulatory coupling between replication and packaging and the a.a. position 27 has a role in this hypothetical mechanism.

These a.a. positions are close to the PA binding site, and the PB1-RNA interaction could be affected by the presence of PA. We examined whether these mutations affect the assembly of RdRp (Figure S1). The assembly of PB1 with PA and PB2 was not affected by these mutations. Moreover, these mutations did not affect the transcription activity, mRNA synthesis from vRNA (Figure 3 and Figure S2). Taken altogether, it is quite likely that amino acids at the positions 27 and 44 are involved in the replication activity, possibly in cRNA promoter recognition with little effects on the transcription activity and the assembly of the RdRp complex.

Recognition of the vRNA promoter depends on the 5′-arm of the promoter and this binding improves the weak binding of RdRp to the 3′-arm of the vRNA promoter (Tiley et al., 1994; Gonzalez and Ortin, 1999a; Jung and Brownlee, 2006). Recognition of the cRNA promoter by RdRp has been shown by the *in vitro* binding of the PB1 subunit with the 5′- and 3′-arms of the cRNA promoter (Gonzalez and Ortin, 1999b). Flexibility within the two uridines of the internal loop of the cRNA promoter required for protein binding in the cRNP complex (Park et al., 2003). Biochemical studies have shown that conformational changes in PB1 of the influenza A virus RdRp lead to the interaction with either vRNA or cRNA (Gonzalez and Ortin, 1999b). Based on previous reports and our findings, the positions 27 and 44 may affect the PB1 structure, resulting in affecting PB1 binding activity to the 3′-arm of the cRNA promoter. Thus, we would propose that these positions in PB1 are important for the replication activity by recognizing the cRNA promoter.

### Conflict of interest statement

The authors declare that the research was conducted in the absence of any commercial or financial relationships that could be construed as a potential conflict of interest.
