# Supplementary material for: The N-terminal region of influenza virus polymerase PB1 adjacent to the PA binding site is involved in replication but not transcription of the viral genome
Source: Front Microbiol. 2013 Dec 18;4:398. doi: 10.3389/fmicb.2013.00398 (PMC3866587; doi:10.3389/fmicb.2013.00398)

Supplementary Methods

RNA analysis by qRT-PCR

MDCK cells were infected with recombinant viruses at the multiplicity of infection (MOI) of 2.5. At 9 hpi, total RNA was isolated by the acid guanidine-phenol-chloroform method. To measure the accumulation levels of viral mRNA, cRNA, and vRNA, quantitative RT-PCR (qRT-PCR) was performed (Kawaguchi and Nagata, 2007; Sugiyama et al., 2009). Viral mRNAs do not have complementary sequence of 5' portion of vRNA since their elongation reaction is interrupted before reaching the 5' terminus of the template to synthesize poly A chains. To selectively synthesize cDNAs of each viral RNA species, 1 μg of total RNAs were subjected to reverse transcription using ReverTraAce (Toyobo) at 42ºC for 1 hr with either (i) oligo (dT)$_{20}$ for synthesizing cDNA from viral mRNA, (ii) 5'-AGTAGAAACAAGGGTATTTTTCTTTA-3', which is complementary to the 3' portion of segment 5 cRNA between nucleotide sequence positions 1540 and 1565 (underline shows the complementary sequence to 3' promoter sequence of cRNA) for synthesizing cDNA from cRNA, or (iii) 5'-GACGATGCAACGGCTGGTCTG-3', which corresponds to segment 5 cRNA between nucleotide sequence positions 424 and 444 for synthesizing cDNA form vRNA. The synthesized single-stranded cDNAs were subjected to real-time quantitative PCR analysis (Thermal Cycler Dice real-time system TP800; TaKaRa) with SYBR Premix Ex *Taq* (TaKaRa) and a set of specific primers, 5'-GACGATGCAACGGCTGGTCTG-3', which corresponds to segment 5 cRNA between nucleotide sequence positions 424 and 444, and 5'-AGCATTGTTCCAACTCCTTT-3', which is complementary to segment 5 cRNA between nucleotide sequence positions 595 and 614. The levels of these RNAs were normalized by the amount of cellular β-actin mRNA measured using specific primers 5'-ATGGGTCAGAAGGATTCCTATGT-3', which corresponds to β-actin cDNA between

nucleotide sequence positions 1363 and 1385, and 5'-GGTCATCTTCTCGCGGTT-3', which is complementary to the β-actin cDNA between nucleotide sequence positions 1567 and 1584.

Immunoprecipitation

MDCK cells were infected with wild type or mutant viruses at MOI of 1. At 7 hpi, cells were lysed by sonication in a buffer containing 50 mM Tris-HCl (pH 7.9), 100 mM NaCl, 30 mM KCl, and 0.1% Nonidet P-40. The lysates were subjected to centrifugation at 16,000 x g at 4°C for 10 min. RNA polymerase complexes in the supernatant fraction were subjected to immunoprecipitation with rabbit anti-PB2 antibody prepared as described previously (Naito et al., 2007) bound to protein A sepharose beads (GE Healthcare) at 4°C, for 2 h. The beads were washed 4 times with the same buffer, and proteins bound to the beads were eluted by boiling them in an SDS-PAGE loading buffer and subjected to 7.5% SDS-PAGE. To detect each viral RNA polymerase subunit, rat anti-PB1, -PB2, and -PA antibodies prepared as described previously (Kawaguchi et al., 2005) and mouse monoclonal antibody against -β-tubulin purchased from Sigma-Aldrich were used for Western blotting analysis.

Supprememental Refferences

Kawaguchi, A., Naito, T., and Nagata, K. (2005). Involvement of influenza virus PA subunit in assembly of functional RNA polymerase complexes. *J Virol* 79, 732-744. doi: 10.1128/JVI.79.2.732-744.2005.

Kawaguchi, A., and Nagata, K. (2007). De novo replication of the influenza virus RNA genome is regulated by DNA replicative helicase, MCM. EMBO J 26, 4566-4575. doi: 10.1038/sj.emboj.7601881.s

Naito, T., Momose, F., Kawaguchi, A., and Nagata, K. (2007). Involvement of Hsp90 in assembly and nuclear import of influenza virus RNA polymerase subunits. *J Virol* 81, 1339-1349. doi: 10.1128/JVI.01917-06

Sugiyama, K., Obayashi, E., Kawaguchi, A., Suzuki, Y., Tame, J.R., Nagata, K., and Park, S.Y. (2009). Structural insight into the essential PB1-PB2 subunit contact of the influenza virus RNA polymerase. *EMBO J* 28**,** 1803-1811. doi: 10.1038/emboj.2009.138.

Supplemental Materials

Table S1. Primers for preparation of PB1 mutants

Position	Forward primer sequence (5’-3’)	Reverse primer sequence (5’-3’)
N16A	CACAAGCTGCTATAAGCACAACTTTC	CTGGCACTTTTAAGAAAAGTAAAGTCG
D27N	CTTATACTGGAAACCCTCCTTAC	GGAAAGTTGTGCTTATAGCATTTTGTGC
D27E	CTTATACTGGAGAGCCTCCTTAC	GGAAAGTTGTGCTTATAGCATTTTGTGC
D27V	CTTATACTGGAGTCCCTCCTTAC	GGAAAGTTGTGCTTATAGCATTTTGTGC
N44I	ATCAGGACACATCAGTACTCAGAAAG	GACAGTATCCATGGTGTATC
N44D	GACAGGACACATCAGTACTCAGAAAG	GACAGTATCCATGGTGTATC
N44Q	CAGAGGACACATCAGTACTCAGAAAG	GACAGTATCCATGGTGTATC

Supplemental Figure Legends

FIGURE S1. Primary transcription activity of mutant viruses

Primary transcription activity of viruses mutated at amino acid position D27 to N, E, and V (A) and N44 to I, D, and Q (B). MDCK cells were infected with mutant viruses at MOI of 2.5 in the presence of 100 μg/ml of CHX. At 9 hpi, the accumulation levels of viral mRNA and vRNA were measured by qRT-PCR, and the amounts of these RNAs were normalized by that of cellular actin mRNA. The transcription activity is represented as a ratio of the amount of viral mRNA to that of vRNA. These results are averages from three independent experiments with standard deviations.

FIGURE S2. Effect of mutations on assembly of viral RNA polymerase complexes

RNA polymerase complexes in infected cells were immunoprecipitated with anti-PB2 antibody. To detect each viral RNA polymerase subunit, anti-PB1, -PB2, and -PA antibodies and antibody against β-tubulin were used for Western blotting analysis. (A) Expression levels of PB2 in infected cells. The amount of PB2 and β-tubulin were determined by Western blotting. (B) The interactions of PB2 and PA with PB1 in infected cells. Immunoprecipitation was carried out with (lanes 1 and 3-12) or without (lane 2) anti-PB2. PB1, PB2, and PA in the immunoprecipitated complex were detected by Western blotting. The ratio of the amount of PB1 to that of PA is shown under each lane.

FIGURE S3. RNA synthesis of D27V virus at various moi

Wild-type virus and D27V mutant virus were infected into MDCK cells at various MOI (0.5, 2.5, 10, and 25). At 9 hpi, the accumulation levels of viral mRNA (A), cRNA (B), and vRNA (C) were measured by qRT-PCR, and the amounts of these RNAs were normalized by that of

cellular actin mRNA. These results are averages from three independent experiments with standard deviations. Asterisk, $p<0.05$; double asterisk, $p<0.01$.

Binh, N. T. et al, Supplemental figure S1

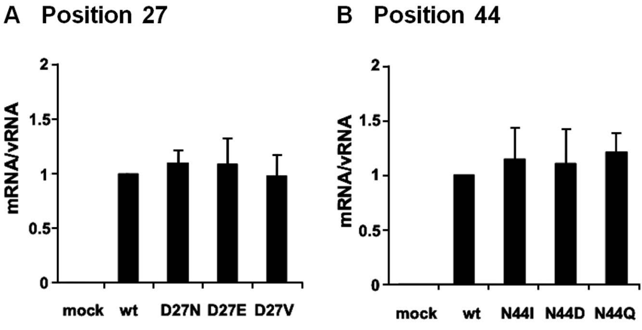

Binh, N. T. et al, Supplemental figure S2

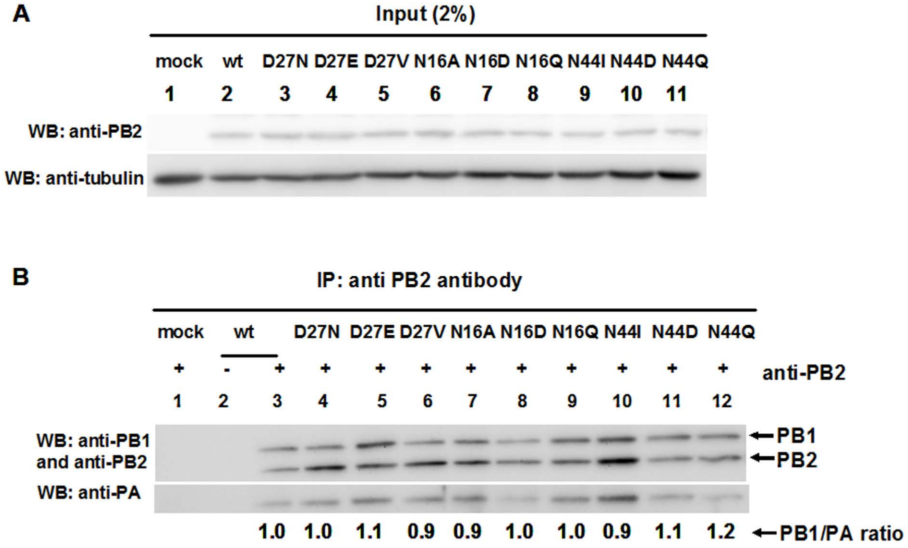

Binh, N. T. et al, Supplemental figure S3

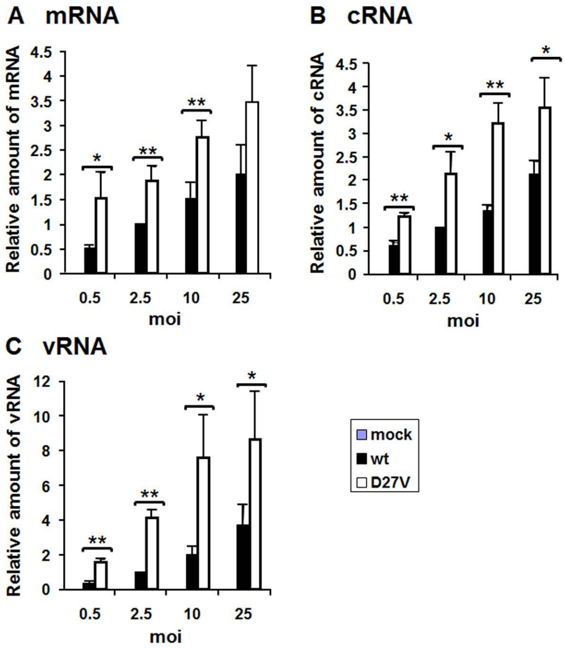

Supplement: Supplementary file 1 [file Presentation1.PDF]
